# An fMRI Study of Audiovisual Speech Perception Reveals Multisensory Interactions in Auditory Cortex

**DOI:** 10.1371/journal.pone.0068959

**Published:** 2013-06-21

**Authors:** Kayoko Okada, Jonathan H. Venezia, William Matchin, Kourosh Saberi, Gregory Hickok

**Affiliations:** Department of Cognitive Sciences, Center of Cognitive Neuroscience, University of California Irvine, Irvine, California, United States of America; Baycrest Hospital, Canada

## Abstract

Research on the neural basis of speech-reading implicates a network of auditory language regions involving inferior frontal cortex, premotor cortex and sites along superior temporal cortex. In audiovisual speech studies, neural activity is consistently reported in posterior superior temporal Sulcus (pSTS) and this site has been implicated in multimodal integration. Traditionally, multisensory interactions are considered high-level processing that engages heteromodal association cortices (such as STS). Recent work, however, challenges this notion and suggests that multisensory interactions may occur in low-level unimodal sensory cortices. While previous audiovisual speech studies demonstrate that high-level multisensory interactions occur in pSTS, what remains unclear is how early in the processing hierarchy these multisensory interactions may occur. The goal of the present fMRI experiment is to investigate how visual speech can influence activity in auditory cortex above and beyond its response to auditory speech. In an audiovisual speech experiment, subjects were presented with auditory speech with and without congruent visual input. Holding the auditory stimulus constant across the experiment, we investigated how the addition of visual speech influences activity in auditory cortex. We demonstrate that congruent visual speech increases the activity in auditory cortex.

## Introduction

In daily conversations, speech is not only heard but it is also seen – auditory speech is typically accompanied by congruent visual speech. Visual cues provide powerful information and aid audition when speech occurs in noisy environments [1,2]. Individuals with early onset hearing loss often rely on visual cues for accurate perception [3] and cochlear implant users demonstrate a greater reliance on visual speech cues than those with normal hearing [4]. This suggests that auditory and visual interactions are an important aspect of speech perception.

Several neuroimaging studies have examined the neural basis of visual speech perception. Lip-reading, without auditory input, activates a network of auditory and language regions such as portions of auditory cortex in the superior temporal gyrus, posterior superior temporal sulcus, inferior frontal gyrus, and premotor cortex [5-10]. Studies that have looked at audiovisual speech have consistently identified the posterior superior temporal sulcus (pSTS) as a site that appears to support audiovisual integration in that it typically shows greater activity for audiovisual speech compared to audio- or visual-speech alone [10-14]. Furthermore, activation in the STS is correlated with behavioral performance on an audiovisual speech integration task [15] and stimulation of the STS interferes with audiovisual speech integration [16], thus demonstrating the region’s causal role in the process.

While the literature clearly indicates that auditory and visual speech interact at higher levels of cortical processing (e.g., STS), what is less clear is how low in the cortical hierarchy multisensory interactions may occur. Several lines of evidence suggest that audio-visual speech interaction may occur at the earliest functional-anatomic stages of cortical processing. For example, research in non-human species has demonstrated multisensory interactions within primary auditory cortex [17-19]. In the speech domain, electrophysiological data indicate that visual speech influences an early temporal stage of auditory processing (between 100–200 msec) [20,21] although it is difficult to pin down the cortical source of such effects.

The present study addresses whether the influence of visual speech extends into core regions of auditory cortex. Although previous studies of silent lip-reading have found activation in lower-level auditory cortical regions [5,6,22] one could argue that such activation reflects auditory imagery rather than cross-sensory interaction. Further, these studies relied on group average activation maps, which can lead to mislocalizations due to averaging error. A stronger test would be to assess whether adding a visual speech signal to an auditory speech signal induces an increase in activity in a functionally defined auditory region in individual subjects. This is what the present study was designed to assess.

## Materials and Methods

### Subjects

Twenty participants (9 female) between 18 and 36 years of age were recruited from the University of California, Irvine (UCI) community and received monetary compensation for their time. The volunteers were right-handed, native English speakers with normal or corrected-to-normal vision, normal hearing, no known history of neurological disease, and no other contraindications for MRI as assessed by self-report. Two subjects were omitted from data analysis due to poor image quality. Written informed consent was obtained from each participant prior to participation in the study in accordance with guidelines from UCI Institutional Review Board, which approved this study.

### Stimuli & Procedure

#### Design overview

In a block design experiment, participants were presented with one of four syllables over headphones (/ra/, /la/, /ma/, /na/). The auditory syllables were paired with matching visual speech, which were videos of mouths articulating the syllables (Audiovisual Condition), or the auditory syllables were presented with a still face (Auditory-Speech Only Condition). Although we assume that any differences in auditory cortex between the audiovisual speech and audio-speech only conditions can be attributed to AV speech integration, it is a possibility that visual motion generally could drive the effect. However, this would not undermine the claim that visual information modulates the auditory system; rather, it would simply generalize it to include non-speech-specific dynamic information as a modulator.

A single block was 15s in length and this was created by concatenating 8 videos with syllables of a single type (/ra/, /la/, /ma/, or /na/) for a total duration of 12.3s (see *Stimuli* section for details) followed by a 2.7s silent period during which subjects made a button press response to indicate which syllable was presented. In each fMRI session, there were 8 Auditory-Speech Only blocks (2 blocks of each syllable) and 8 Audiovisual blocks. In addition, two rest trials (scanner noise presented with a still face) 15s in length were randomly interspersed throughout each session. Presentation order of the blocks was randomized for each subject with respect to speech sound category (/ra/, /la/, /ma/, /na/) and stimulus condition (Audiovisual or Auditory-Speech Only).

We also included an auditory cortex localizer scan, which consisted of broadband noise amplitude modulated at 8 Hz. The purpose of this scan was to provide an independent localizer of an auditory cortex region of interest within which we could then examine the effects of audiovisual stimulation in the main experiment.

#### Stimuli

Audiovisual stimuli were recorded in a quiet, well-lit room. Digital videos of the speaker were recorded (30 frames/s) while audio was recorded on a separate microphone and digitized at 44.1 kHz. Audio information was also captured by the built-in microphone on the digital video camcorder. Audio and video files were synced manually by aligning the auditory waveform with the built-in audio recording. Thus, the natural timing of audiovisual speech information was preserved in all stimuli.

During recording, a talker produced approximately 20 samples of each syllable using natural timing and intonation, and pausing briefly between each sample over the course of a single continuous session. A set of 12 tokens were chosen for each syllable based on informal evaluation of loudness, clarity and quality of the audio recording. For each token, a 46-frame (1.533s) video was extracted from the continuous recording with the visual speech information centered in time. The corresponding auditory information was extracted and synced as described above (mean auditory syllable length = ~ 400ms). The fundamental frequency (f0) was estimated for each auditory stimulus and all stimuli were then normalized to the overall mean f0 (89.7 Hz, sd = 1.54 Hz) and matched for root-mean-square power.

Stimulus blocks were created by concatenating eight videos from within a speech sound category. A single token was never repeated within a block and the order of tokens within a block was chosen pseudorandomly such that each token appeared an equal number of times across all blocks. For the Auditory-Speech Only condition, the audio was extracted and presented with a 12.3s clip of a still frame of the talker’s face at rest.

#### Procedure

The experiment started with a short exposure session to familiarize subjects with the task and learn the mapping between syllable and button box. Subjects were scanned during the exposure session to ensure they could comfortably hear the stimuli through the scanner noise, and to acclimatize them to the fMRI situation. Following 9 experimental sessions, the study ended with a localizer scan which consisted of 10 cycles of amplitude modulated broadband noise (8 Hz) alternating with rest (scanner noise) in 15s intervals. All stimuli were presented with MR compatible headset and stimulus delivery and timing were controlled using Cogent software (http://www.vislab.ucl.ac.uk/cogent_2000.php) implemented in Matlab 6 (Mathworks, Inc, USA).

### Scanning parameters

MR images were obtained in a Philips Achieva 3T (Philips Medical Systems, Andover, MA) fitted with an 8 channel RF receiver head coil, at the high field scanning facility at the University of California, Irvine. Images during the experimental sessions were collected using Fast Echo EPI (sense reduction factor=2.4, matrix=112x112mm, TR=3.0s, TE=25ms, size=1.95x1.95x2mm). After the functional scans, a high resolution anatomical image was acquired with an MPRAGE pulse sequence in axial plane (matrix=256x256mm, TR=8ms, TE=3.7ms, flip angle=8°, size=1x1x1mm).

### Data Analysis

We utilized both a standard whole brain group analysis to replicate previous studies and an individual subject, ROI-based approach to allow us more power in assessing our specific hypothesis.

Data preprocessing and analyses were performed using AFNI software (http://afni.nimh.nih.gov/afni). First, motion correction was performed by creating a mean image from all of the volumes in the experiment and then realigning all volumes to that mean image using a 6-parameter rigid-body model [23]. The images were then smoothed with an isotropic 6 mm full width half maximum (FWHM) Gaussian kernel. The anatomical image for each subject was coregistered to his/her mean EPI image.

First level analysis was performed on the time course of each voxel’s BOLD response for each subject using AFNI software [24]. Regression analysis was performed with regressors created by convolving the predictor variables representing the time course of stimulus presentation with a standard hemodynamic response function [25]. The three regressors used in the estimation of the model were the following: Audiovisual condition, Auditory-Speech Only condition, Still Faces. An additional 6 regressors representing the motion parameters determined during the realignment stage of processing were entered into the model. For the auditory localizer session, one regressor associated with presentation of noise was entered into the model along with the 6 motion regressors. An F statistic was calculated for each voxel and statistical parametric maps (SPMs) were created for each subject. To test specific hypotheses, linear contrasts were also performed and T-statistics were computed at each voxel to identify regions significantly activated in the Audiovisual condition compared with the Auditory-Speech Only condition. To facilitate group analyses, functional maps for each participant were transformed into standardized space and resampled into 2mm^3^ voxels using the MNI template, http://www.bic.mni.mcgill.ca/brainweb/). Second-level analysis was then performed on the linear contrasts of the parameter estimates from each participant, treating participants as a random effect and voxel-wise t-tests were performed. Group analysis was thresholded at q<0.05 using the false discovery rate correction.

### ROI Selection & Analysis

**Figure 1:**
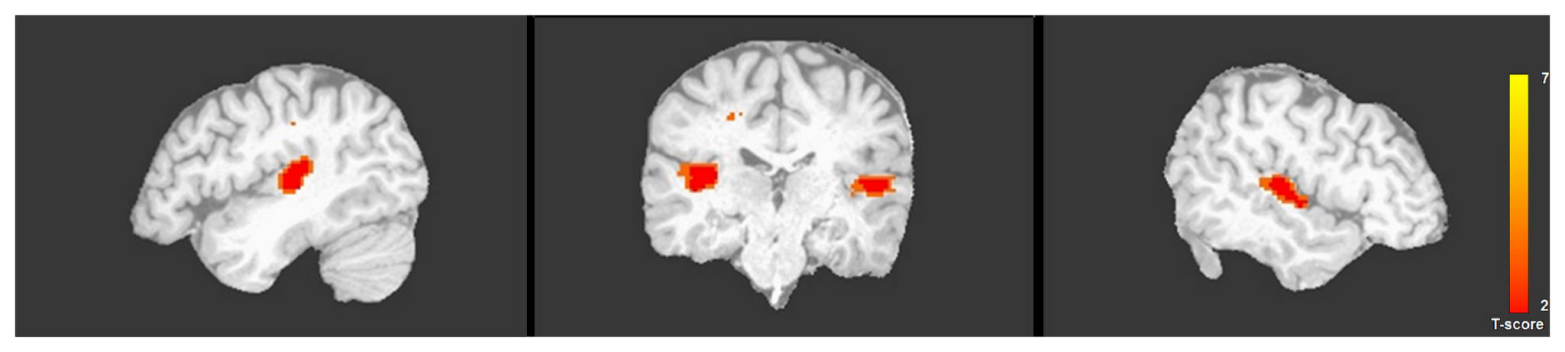
A representative subject illustrating activation in auditory cortex in the localizer session (p<0.001).

**Figure 2:**
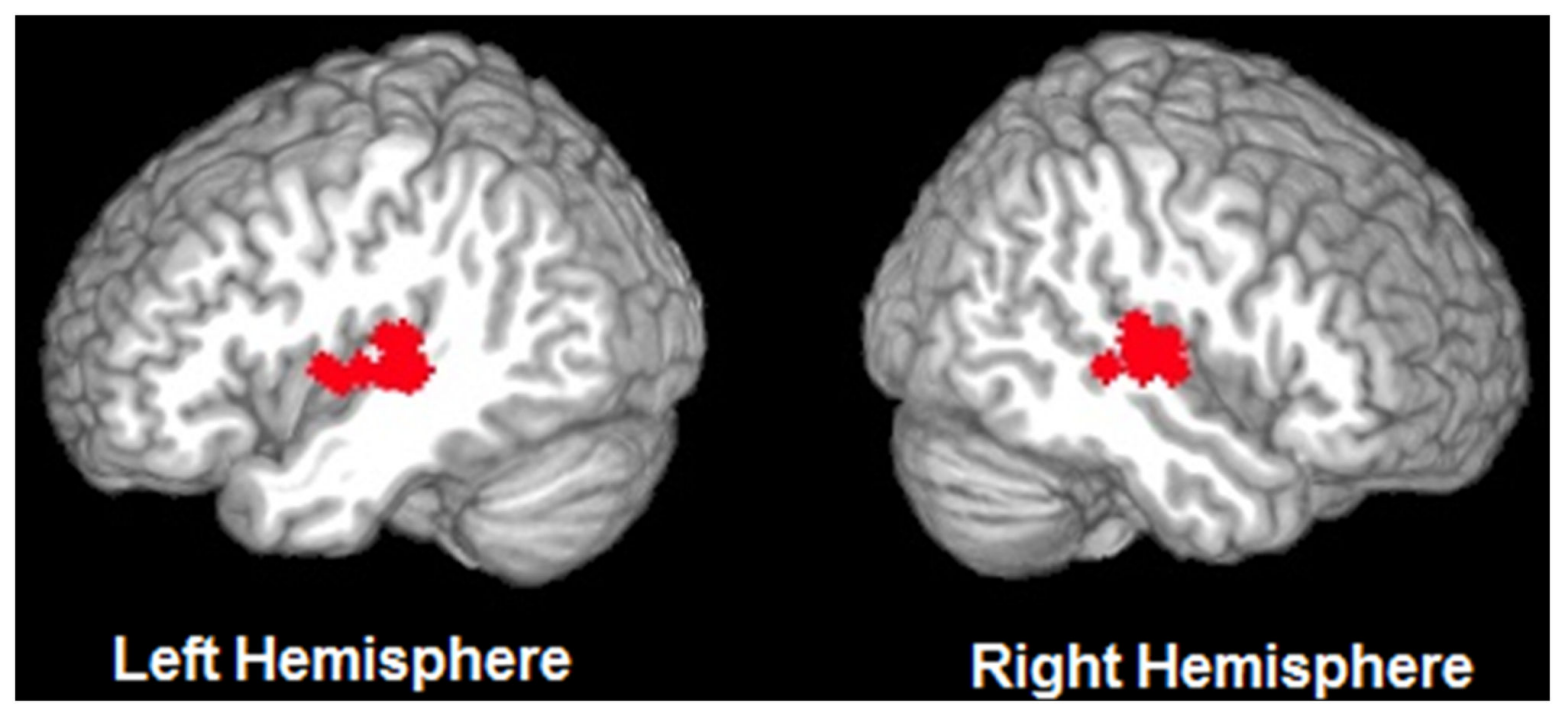
Displays the ROI selected in each subject (N=14) overlaid on a surface-rendered template brain. Voxels were selected using a functional localizer.

In each subject, auditory cortex voxels of interest were functionally identified using the localizer session. Figure 1 illustrates activation in the localizer session in a representative subject, and Figure 2 displays the ROIs selected from each subject. Fourteen subjects had auditory cortex activity in both hemispheres (p<.001), and were included in the timecourse analysis. Using the contrast Noise > Rest (p<.001), the peak voxel in each hemisphere in auditory cortex was identified. Using this peak, a focal cubic ROI was drawn for each subject that included 5x5x5 voxels (i.e., +/- 2 voxels in each direction from the peak). The mean MNI coordinates of the peaks in each hemisphere were, LH = [-52-23 5], RH = [55-18,5]). Within the 5x5x5 voxel ROI only voxels that were significantly activated in the auditory cortex localizer were included in subsequent analyses to ensure that the analysis involved voxels that were highly responsive to auditory stimulation (average number of voxels: LH=87 voxels, RH=84 voxels). Unsmoothed data from the nine experimental sessions within the separately defined ROIs were used for timecourse analysis. First, data from each participant were normalized by transforming the voxel values into z-scores across time points. Then the mean response for each condition at each time point was calculated across subjects and the averaged activation was represented by 5 timepoints per condition. To assess the magnitude of the effect in auditory cortex from the normalized time series, difference scores were calculated at each timepoint by subtracting the minimum z-score of each condition from the z-score at each timepoint. These values, excluding the first timepoint were then averaged across the remaining timepoints for each condition in each hemisphere in each subject. The beginning timepoint values did not differ across conditions or hemispheres (left hemisphere: Auditory Speech Only =0.08, Audiovisual =0.1; right hemisphere: Auditory Speech Only =0.1, Audiovisual Condition=0.09). A 2 x 2 repeated measures ANOVA was performed on these values, entering condition and hemisphere as factors.

**Figure 3:**
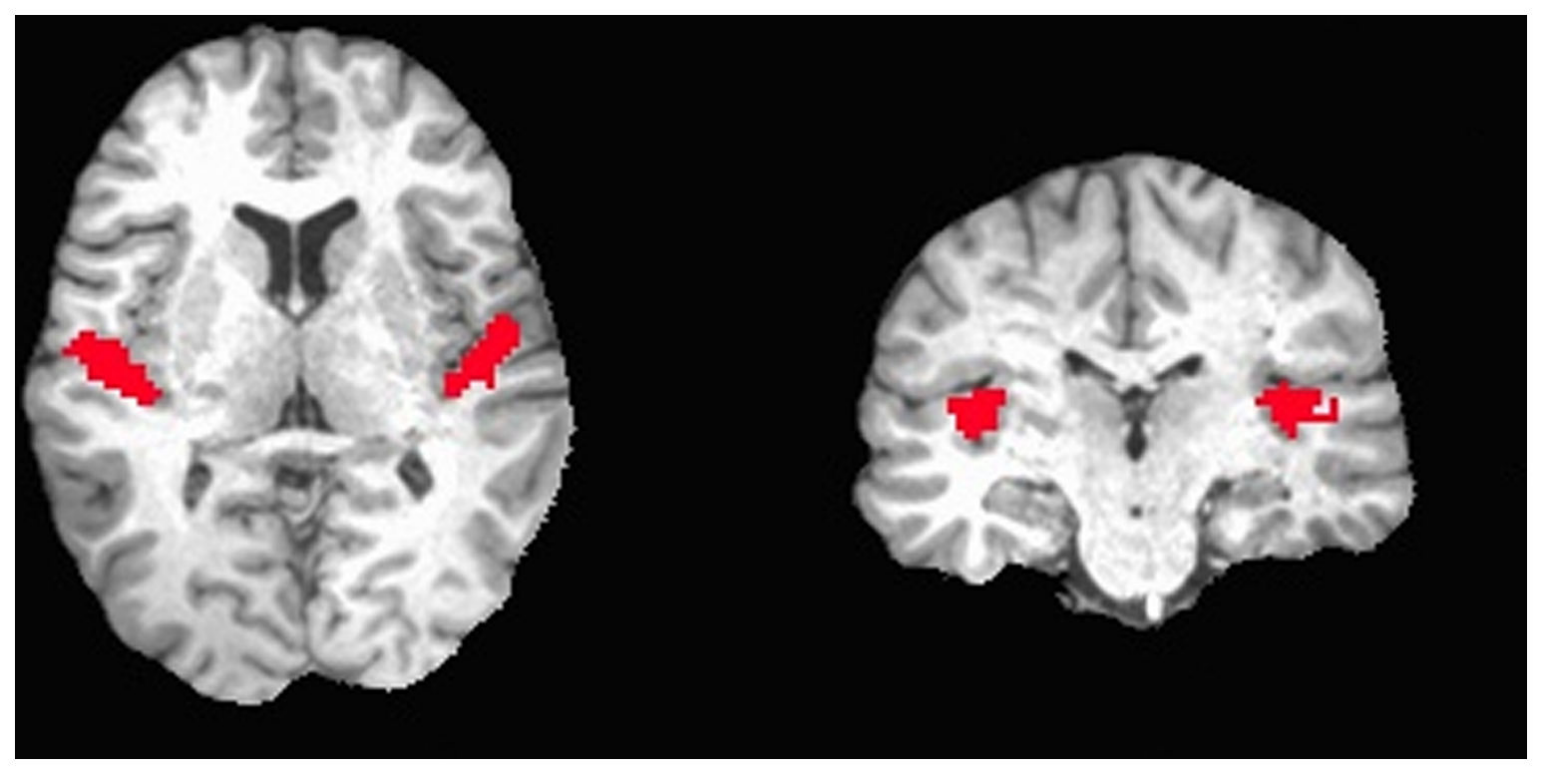
A representative subject illustrating voxels selected using an anatomically defined ROI.

In addition to selecting voxels in auditory cortex using a functional definition, an additional analysis was performed on voxels selected using an anatomically defined ROI. First, we used a cytoarchitectonic probability map (included in the AFNI software package) to create a new auditory cortex mask that only included cytoarchitectonic areas Te1.0, Te1.1 and Te1.2 which covers Heschl’s gyrus [26]. This mask was transformed from Talairach space into native space for each subject and each mask was visually inspected to ensure it covered Heschl’s gyri in both hemispheres (see Figure 3). Voxels contained in this mask were extracted and timecourse analysis was performed as described above. Because an anatomical definition was used for voxel selection, all 18 subjects were included in this analysis regardless of whether or not they had significant activation in auditory cortex in the functional localizer session.

## Results

### Whole brain group analysis results

**Figure 4:**
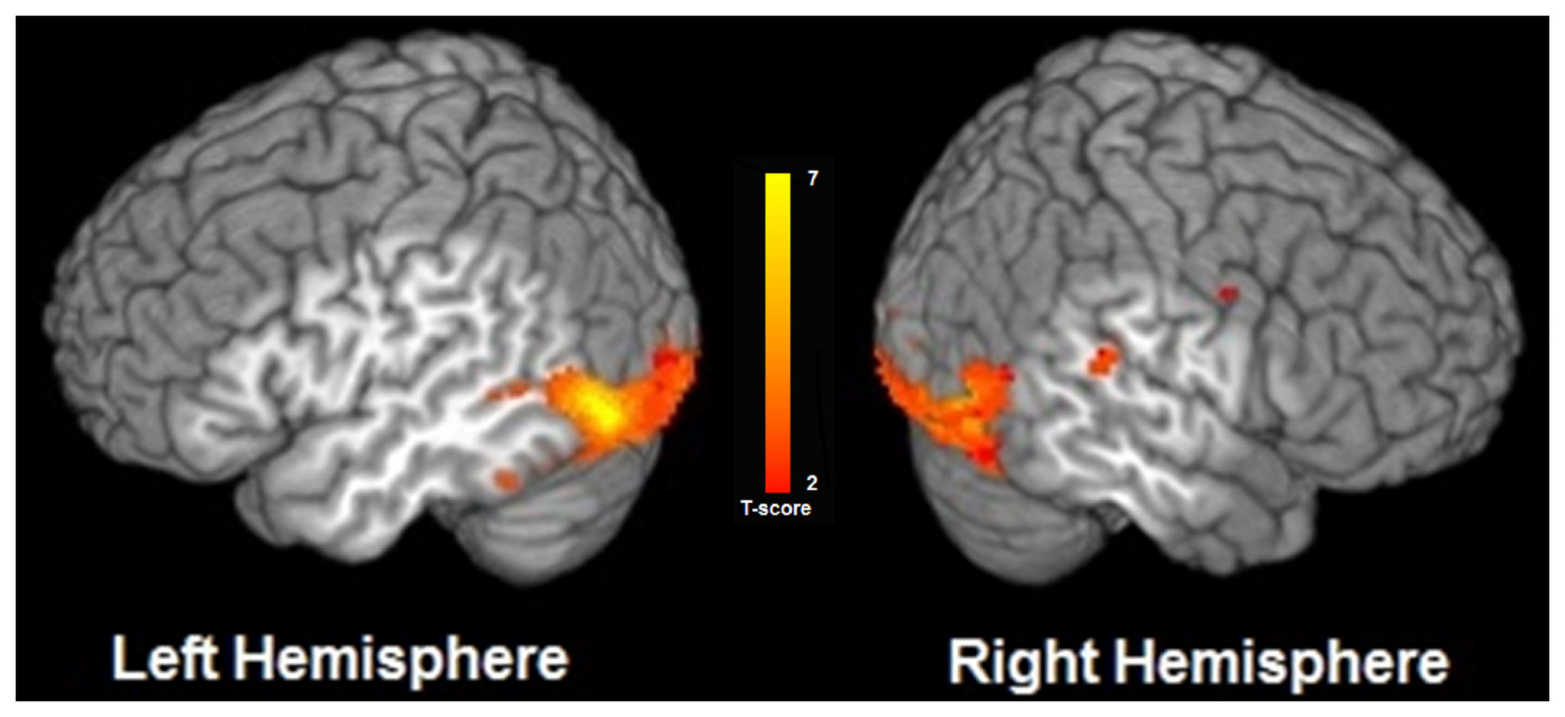
Group map illustrating regions significantly activated in the Audiovisual > Auditory-Speech Only contrast. Group activation map (N=18, false discovery rate q <0.05) overlaid on a surface-rendered template brain.

In group analysis, a contrast of the Audiovisual Condition compared with the Auditory-Speech Only condition (AV>A) yielded activation in left posterior superior temporal sulcus (pSTS), left middle temporal gyrus and right superior temporal gyrus (q<0.05, FDR corrected). We also found significant activation in several other regions such as bilateral inferior and middle occipital cortices, bilateral anterior cingulate, left insula and left superior frontal gyrus. Figure 4 illustrates regions significantly activated in the Audiovisual condition compared to the Auditory-Speech Only condition, and Table 1 provides a summary of the MNI coordinates of the center of mass of activated clusters in this contrast. Notably, in the group-level analysis we did not find any significant activation in auditory cortical regions in the supratemporal plane, i.e., in and around the auditory core on the dorsal surface of the temporal lobe. To explore possible cross-sensory interactions in these auditory regions lower in the cortical hierarchy, we employed an ROI approach in individual subjects.

**Table 1:** Regions activated in the contrast Audiovisual > Auditory Speech Only.

Region						Voxels	CM x	CM y	CM z
Right Hemisphere									
	Middle Occipital Gyrus					2587	36.1	-76	-8.5
	Insula, Superior Temporal Gyrus					132	56	-30.7	17.8
	Amygdala					70	20	-4.6	-16.3
	Precentral Gyrus					16	52.5	-2.6	40.1
Left Hemisphere									
	Middle Occipital Gyrus					2042	-35.5	-81.3	-6.8
	Anterior Cingulate					148	-4.3	51.3	-6.7
	Fusiform Gyrus					46	-45	-45.6	-20.9
	Middle Temporal Gyrus/ Superior Temporal Sulcus					11	-47.2	-45.1	4.7
	Superior Frontal Gyrus					11	-16.3	56	18.7

MNI coordinates of the center of mass in activated cluster for the contrast of Audiovisual > Auditory-Speech Only in the group analysis (N=18, cluster threshold=10 voxels, false discovery rate q < 0.05)

### ROI analysis results

**Figure 5:**
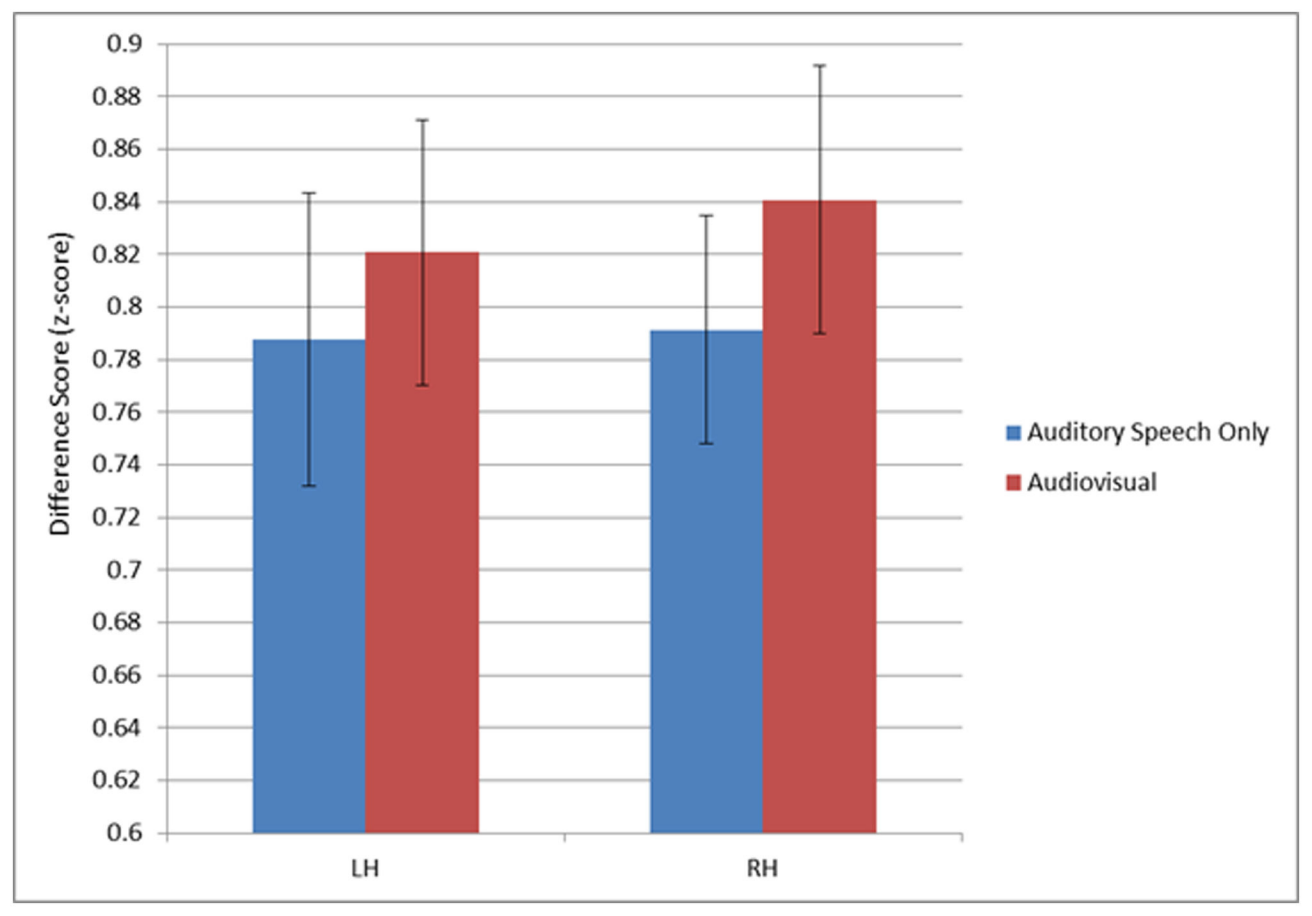
Graph showing the mean difference scores with 95% confidence intervals from voxels in auditory cortex. There was a significant main effect of condition (N=14, p=0.02). Mean values are computed as the average of the z-score at each timepoint minus the minimum z-score for each condition in each hemisphere.

The first ROI analysis was performed on the voxels extracted from auditory cortex using the functional localizer scan. We performed a repeated measures ANOVA treating condition and hemisphere as factors. The ANOVA revealed a significant main effect of Condition, F(1,13)=6.993, p=0.02. There was greater activity in the Audiovisual condition compared to the Auditory-Speech Only condition (see Figure 5). That is, addition of congruent visual speech to auditory speech produced an increase in signal amplitude in auditory cortex. The main effect of Hemisphere was not significant (F(1,13)=3.346, p=0.090) and the Hemisphere x Condition interaction was not significant, (F(1,13)=3.482, p=0.085) although trended toward more activation in the right hemisphere primarily attributable to the Audiovisual condition. A second repeated measures ANOVA was also performed on voxels extracted using an anatomically defined mask, and analysis yielded similar results with significantly greater activation in the Audiovisual condition compared to the Auditory-Speech Only condition, although only in the left hemisphere.

## Discussion

Consistent with previous research, our whole brain group analysis revealed that audiovisual speech perception activated posterior superior temporal sulcus (pSTS) bilaterally to a greater extent than auditory speech alone. Auditory regions lower in the cortical hierarchy in the supratemporal plane did not show the same effect in this whole brain analysis. However, the response of these areas was examined more closely using a functionally defined ROI approach in individual subjects, which revealed that visual speech does modulate activity in lower cortical stages of the auditory processing hierarchy.

A number of previous studies have reported effects of visual stimulation in auditory cortex including studies of lipreading (i.e., visual speech without auditory stimulation) [5,27], electromagnetic and hemodynamic studies of audiovisual speech integration [20,21,28] and audiovisual integration of non-phonemic information such as emotional or gender information [29-31]. However, none of these unambiguously localize the effects to early stages in the cortical processing hierarchy. The most robust anatomical localization of audiovisual integration across studies is the STS, which represents a fairly high-level auditory or even multisensory processing region [14,32,33]. Studies of lipreading have identified activation effects in the supratemporal plane, which could represent cortical stage of influence lower in the processing hierarchy. However, such effects could be interpreted as auditory imagery. Electromagnetic studies can localize the effects of audiovisual integration in time, but cannot unambiguously localize them anatomically, as effects occurring in the 100-200 msec time window could reflect activity in fairly high-level processing regions.

The present study sought to circumvent these ambiguities in interpretation by using a low-level functional localizer to define ROIs in and immediately surrounding Heschl’s gyrus (the auditory core on the dorsal surface of the temporal lobes) and by assessing whether the addition of visual speech to auditory speech further boosts activation levels within the ROIs (thus minimizing the possibility of auditory imagery driving the effects). As such, our results suggest that auditory and visual interactions occur not only in high-level language-related regions such as pSTS, as has been documented repeatedly, but that interactions can occur lower in the cortical processing stream. This finding is consistent with work in both nonhuman primates and other species suggesting that there are direct anatomical connections between primary cortices and that multisensory responses can be observed in unimodal regions [19,34].

There may be several routes for visual speech to exert influence on auditory processing in and around the auditory core. First, there could be inputs from multisensory regions such as pSTS, with higher level processing influencing low level processing. Second, activity in the auditory cortex could be directly modulated by input from visual cortex. For example, in non-human primates, anatomical connections between primary auditory cortex and primary visual cortex have been demonstrated [35] and in humans, a recent neuroimaging study using effective connectivity analysis demonstrated that activity in auditory cortex is modulated by both direct connections between visual and auditory cortex, as well as indirect connections through superior temporal sulcus [36]. Third, activity in auditory cortex may be influenced by multisensory interactions stemming from sub-cortical regions [37,38]. In other non-human species such as rodents and cats, multisensory interactions have been demonstrated in early processing areas such as the superior colliculus and primary auditory cortex [37]. In ferrets, it has been demonstrated that visual inputs to auditory cortex can modulate processing of auditory stimuli [39]. In the present experiment, it may be that all of these routes are used to exert influence on auditory activity.

It seems clear that multisensory interactions occur at multiple levels in the processing hierarchy [21,36,40]. Our current study demonstrates that in humans, visual speech can exert influence on heard speech in lower cortical stages of auditory processing. One possible function of such influence is top-down predictive coding to help constrain the appropriate speech sounds as auditory signals are processed [21]. The computational mechanism of predictive coding is topic of current investigation. In the context of motor control models, predictive coding (forward models) is typically modeled as an inhibitory or suppression signal, such that when the predicted sensory feedback is realized the two signals roughly cancel [41]. Some authors have adapted this mechanism for predictive coding that does not necessarily involve the motor system. For example, Friston points out that top-down prediction could be instantiated as suppression signals, with only error signals (the difference between top-down prediction and driving inputs) being propagated forward in the cortical hierarchy [42]. An alternative is that top-down prediction could be modeled more like attentional gain control mechanisms [43]. Or, a somewhat different approach to thinking about cross-modal enhancement of sensory processing is via an oscillation phase resetting mechanism: it has been suggested that for multimodal signals, timing or stimulus onset information from one modality could reset the phase of intrinsic oscillations thus maximizing synchrony between intrinsic and stimulus generated neural signals [44].
